# Topical and Ingested Cooling Methodologies for Endurance Exercise Performance in the Heat

**DOI:** 10.3390/sports6010011

**Published:** 2018-02-02

**Authors:** Russ Best, Stephen Payton, Iain Spears, Florence Riera, Nicolas Berger

**Affiliations:** 1 School of Social Sciences, Humanities & Law, Teesside University, Middlesbrough TS1 3BX, UK; N.Berger@tees.ac.uk; 2 Centre for Sports Science and Human Performance, Waikato Institute of Technology, Hamilton 3288, New Zealand; 3 City of Glasgow College, Glasgow G4 0RF, UK; stephen.payton@cityofglasgowcollege.ac.uk; 4 Pro-Football Support Ltd., Huddersfield HD7 5BQ, UK; iain.spears@gmail.com; 5 Laboratoire ACTES–EA 3596, Université des Antilles et de la Guyane, 97157 Pointe à Pitre, France; florence.riera@gmail.com; 6 Laboratoire LEPSA–EA 4604, Université de Perpignan, 66120 Font Romeu, France

**Keywords:** cooling, environment, endurance performance, thermal comfort, thermal sensation, temperature

## Abstract

This systematic review and meta-analysis aimed to assess studies which have investigated cooling methodologies, their timing and effects, on endurance exercise performance in trained athletes (Category 3; VO_2max_ ≥ 55 mL·kg·min^−1^) in hot environmental conditions (≥28 °C). Meta-analyses were performed to quantify the effects of timings and methods of application, with a narrative review of the evidence also provided. A computer-assisted database search was performed for articles investigating the effects of cooling on endurance performance and accompanying physiological and perceptual responses. A total of 4129 results were screened by title, abstract, and full text, resulting in 10 articles being included for subsequent analyses. A total of 101 participants and 310 observations from 10 studies measuring the effects of differing cooling strategies on endurance exercise performance and accompanying physiological and perceptual responses were included. With respect to time trial performance, cooling was shown to result in small beneficial effects when applied before and throughout the exercise bout (Effect Size: −0.44; −0.69 to −0.18), especially when ingested (−0.39; −0.60 to −0.18). Current evidence suggests that whilst other strategies ameliorate physiological or perceptual responses throughout endurance exercise in hot conditions, ingesting cooling aids before and during exercise provides a small benefit, which is of practical significance to athletes’ time trial performance.

## 1. Introduction

Heat exposure imposes perceptual and physiological demands on athletes that can be attenuated by interventions; however, the precise timing and best method of administration for these remain unclear. Cooling strategies applied before (precooling) and during (percooling) exercise have been shown to ameliorate deleterious symptoms experienced whilst exercising in the heat [1]. Strategies to attenuate these factors are of importance given the increasingly global nature of elite endurance sports, and the consequent scheduling demands placed upon athletes who often arrive at events with little time for heat-acclimatisation.

Independent of the attenuation of physiological symptoms [2,3,4], improving perceptual symptoms of heat exposure (e.g., thermal comfort and strain) has been shown to improve exercise and cognitive performance [5,6]. Recently, subjective measures of athlete wellbeing/fatigue have been shown to be more reliable and sensitive than objective indices in predicting performance, better reflecting acute and chronic training stresses [7]. These findings highlight the importance of how an athlete feels in determining performance outcomes. This is likely due to an improved ‘interoceptive state’ [8] whereby athletes’ physiological condition, motivation [8,9], and perception of effort [9] are positively affected due to sensations that are perceived to be beneficial in maintaining homeostasis or facilitating task completion [8,10]. Hence cooling methodologies that display no physiological effect but improve psychological condition may still be of value to the athlete with respect to performance.

The emergence of contemporary ingested precooling and percooling strategies, such as ice slurry and menthol mouth swilling, and ice slush ingestion [4,5,11,12,13] have shown improvements in endurance capacity and performance, whilst also being highly practical [14]. Topical cooling strategies are also well established [1,14] and have been shown to be effective (small to moderate effects) when applied before or during an exercise bout [1]; however, implementation may be impractical outside of the lab [14]. These strategies may be of most use in elite endurance athletes [5,15,16] who face extended periods of heat exposure, often on successive days. Therefore, the following meta-analysis assesses the evidence for the performance effects of ingested and topical precooling and percooling strategies on trained endurance athletes exercising in the heat. A secondary aim was to explore the magnitude of effect different cooling strategies and timings have upon physiological and perceptual outcomes that pertain to endurance performance.

## 2. Materials and Methods

Articles investigating ingested and topical precooling and percooling strategies or a combination of both methodologies, were sourced from six online databases (BioMed Central, CINAHL, PlosOne, PubMed, SPORTDiscus, and Web of Science). Reference lists of selected articles were also checked for relevant articles. Where full texts were not available from the university’s library, copies were requested from the British Library. Four search terms were constructed by combining one of four independent variable search terms with a dependent variable term, using the Boolean operator AND. Each search term was performed in each database. The independent variable terms were as follows: Precooling OR pre-cooling OR “pre cooling” OR cooling; Cooling AND Exercise; “Cooling during Exercise”; Cooling AND “during Exercise”. The dependent variable term read: “Time Trial” OR “Time to Exhaustion” OR Power Output OR “Rating of Perceived Exertion”.

Included results were limited to full-text journal articles written in English, published prior to 11 May 2017. Article titles and abstracts of search results were screened in accordance with exclusion criteria; full texts of the remaining articles were obtained and screened. Within- or between-subjects, repeated measures crossover, and randomised controlled trial designs in healthy adults (male only or male and female participants, absent of spinal cord injury, within Performance Level 3 or better [17] (pooled VO_2max_ ≥ 55 mL·kg·min^−1^)) conducted in temperatures ≥28 °C were considered for inclusion. This temperature range was selected because it is equal to or exceeds the human thermoneutral zone [18,19] and is greater than the temperature at which the rate of metabolic heat production exceeds the rate of thermal transfer (25 °C [20]). A trained participant pool was selected to increase the reliability of the included studies [21,22,23] and support the translation of our findings into practice by athletes or support personnel. Ingested (cold water, ice slurry, menthol) and topical (cooling garments, ice packs, sprays) precooling, percooling, and combined (precooling and percooling) methodologies were assessed. Only individual, non-ultra-endurance exercise modalities were considered (cycling, running, swimming, and triathlon completed within the confines of standardised competitive distances or training for such events). Outcome measures had to relate to aerobic exercise performance, with perceptual or physiological measures of heat also being reported. Studies were included on the condition that two reviewers (R.B. and S.P.) agreed that they met the inclusion criteria. If there was disagreement between reviewers, then a third reviewer (N.B.) was consulted.

**Figure 1:**
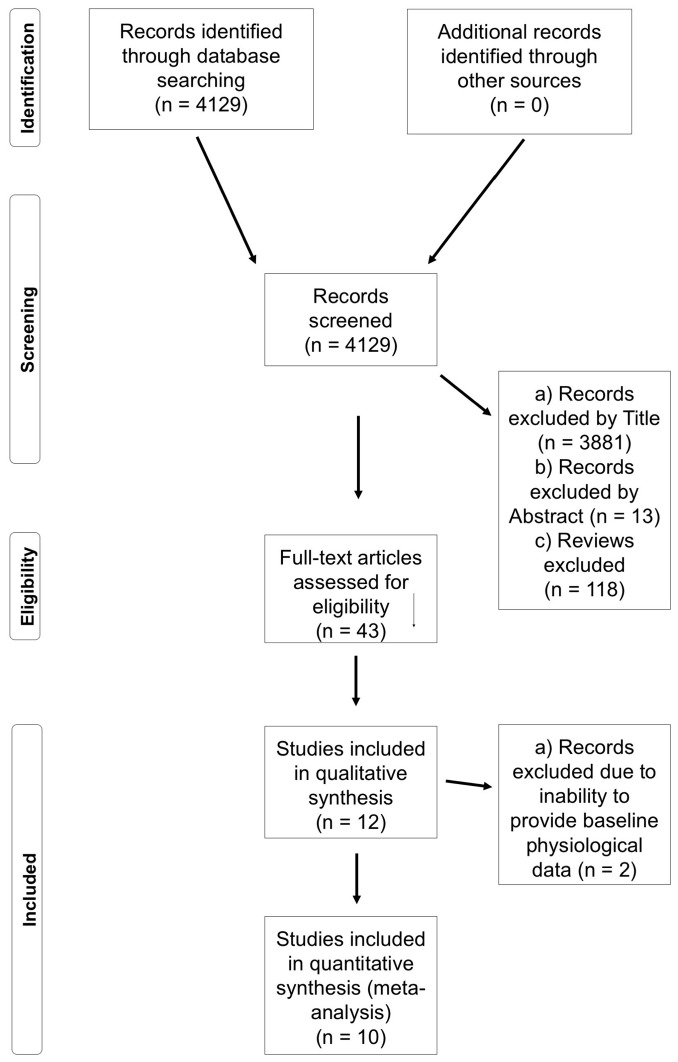
Flow chart to depict the study search, screening, and inclusion process.

The initial series of searches yielded 4129 results; after screening titles, abstracts, and repeats, 43 full texts were obtained. These texts were reduced to 16 in accordance with the inclusion and exclusion criteria, which were then reduced to 11 upon further review (Figure 1). Data were obtained from authors prior to meta-analysis. Means and standard deviations from each study were calculated and used to quantify effect sizes (ESs) with accompanying 90% confidence intervals (CIs) using specialist software (Review Manager Version 5.3., The Cochrane Collaboration, Copenhagen, Denmark), ESs are described as follows: Trivial: 0.0–0.2; Small: 0.2–0.6; Moderate: 0.6–1.2; Large: 1.2–2.0; Very Large: 2.0–4.0; and Extremely Large: ≥4.0 [24].

Methodologies employed, as well as perceptual and physiological outcomes for each study are detailed in Table 1. Methodological quality of studies was assessed using the previously validated PEDro Scale [25]. A publication bias is acknowledged given the trained nature of the participants studied [1,3] and the emphasis placed upon the practicality of the strategies under review. Heterogeneity was assessed using Cochran’s *Q* test (p < 0.05) and I^2^ (low ≥25%, moderate ≥50%, high ≥75%) for all studies.

## 3. Results

Eleven studies, with a combined sample of 101 athletes (310 observations), were included for meta-analysis; participants had a pooled VO_2max_ of 63.09 ± 4.55 mL·kg^−1^·min^−1^. Of the 10 studies included for meta-analysis 3 employed precooling, 5 percooling, and 3 combined intervention timings; with 5 utilising topical, 7 ingested, and 3 combined cooling methodologies. PEDro Scoring revealed all studies to be of high quality (PEDro Score 6). We observed non-significant, low heterogeneity (*Q* = 11.06, (p = 0.92), I^2^ = 0%) across all studies. Raw differences (Δ performance; seconds) in time trial performances are presented in Table 2.

### 3.1. Timing of Cooling Methods

**Figure 2:**
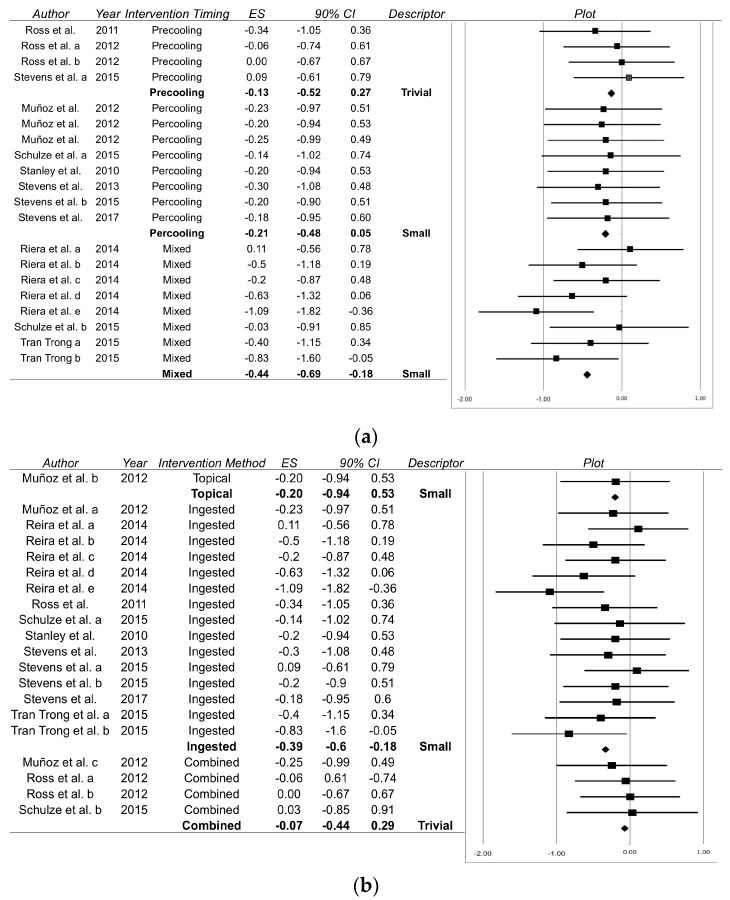
Forrest plot displaying the difference in time trial performance between the experimental group and control group for each individual case; (**a**) when employing differing cooling strategies and (**b**) when administering strategies at different time points. ES: Effect Size; ■: Mean response for an individual study; ♦: Pooled effect size for included studies.

Mixed timings (a combination of precooling and percooling) were found to be the most effective timing with respect to time trial performance (Figure 2a; Effect Size = −0.44; 90% Confidence Interval −0.69 to −0.18), with precooling and percooling demonstrating trivial and small effects, respectively (Figure 2a). Power output was trivially improved by precooling (0.17; −0.18 to 0.52) and percooling (0.16; −0.40 to 0.73), with no power output data reported for mixed timings. Percooling (−0.37; −0.65 to −0.10) and precooling (−0.42; −0.93 to 0.10) strategies demonstrated small reductions in rectal temperature, whereas mixed methods elicited a near moderate decrease (−0.59; −0.90 to 0.28). Effects upon perceptual measures were varied. Thermal comfort and sensation were found to be most receptive to percooling (1.29; −0.82 to 1.76 and −0.60; −1.51 to 0.31, respectively) but presented broad confidence intervals. Small beneficial reductions in rating of perceived exertion (RPE) were found following percooling (−0.39; −0.70 to −0.08) and mixed (−0.48; −0.75 to −0.22) timings, whereas precooling trivially influenced RPE (0.17; −0.18 to 0.52).

### 3.2. Application of Cooling Methods

Small improvements in time trial performance (−0.39; −0.60 to −0.18) and power output (0.22; −0.22 to 0.66) were seen following ingested cooling methodologies (Figure 2b). Similarly, small effects were also observed in topical strategies for time trial performance (Figure 2b) and power output (0.34; −0.40 to 1.08), yet combined strategies showed trivial effects in both parameters (see Figure 2b for time trial performance; power output: 0.02; −0.39 to 0.44). Rectal temperature was most sensitive to ingested (−0.47; −0.68 to −0.26) methodologies, presenting moderate effects. This was not supported by measures of thermal comfort, for which large reductions were found following application of topical strategies (−1.35; −2.18 to −0.51). Thermal sensation was most sensitive to combined strategies (−0.36; −1.25 to 0.53). Topical (−0.50; −1.03 to 0.04) and ingested (−0.41; −0.61 to −0.20) methodologies induced small beneficial effects upon RPE, with trivial effects found when combined strategies were applied (−0.13; −0.53 to 0.27).

## 4. Discussion

This meta-analysis aimed to assess the effects of practical precooling and percooling strategies applied to trained endurance athletes exercising in hot environmental conditions. Our main finding was that combining precooling and percooling timings has a cumulative beneficial effect upon endurance time trial performance, compared to when precooling and percooling are implemented in isolation (Figure 2a). Our secondary finding was that ingested cooling methods outperform topical, or a combination of methods, suggesting method of delivery affects the performance enhancing capabilities of cooling interventions (Figure 2b). Therefore, when competing in the heat, we recommend ingesting cold liquids or ice slurries before and during competition.

This contrasts the conclusions of recent analyses that have suggested precooling and percooling impart similar performance benefits [1], and that combined or topical cooling methodologies are of most value to the athlete [26]. Mixed timings show a greater effect in the reviewed studies (Figure 2a) than when precooling and percooling are performed independently, whereas Bongers et al. [1] found similar effects between precooling and percooling (0.44 and 0.40, respectively). Bongers et al. [1] state an absence of combined cooling timing research (mixed timings) but suggest that implementation of such strategies may prove effective. Our analysis clearly supports their suggestions. This may be a dose response relationship, as combined timings typically include a greater number of cooling exposures than when precooling or percooling is conducted in isolation. The timing of cooling exposures may also have physiological or practical implications, for example, possible interference with warm-up or call room procedures; it may be prudent for event organisers to maximise cooling opportunities in thermally challenging events. This may improve athletes’ performances, preserve athlete health [27], and reduce the prevalence of heat associated illnesses during such events [28].

The clear difference in findings between our and other reviews may also be attributed to a difference in what authors consider ‘performance’. We chose to review the effects of cooling on time trial performance, as this is a meaningful measure for endurance athletes. Other reviews [1,29] have grouped endurance outcomes (time trial performance, distance completed, time to exhaustion, power output, etc.) under a broad definition of performance. Whilst cooling may produce similar effect statistics on differing endurance parameters, the tests implemented assess differing endurance functions (capacity vs. performance) [22] and, importantly, display differing levels of repeatability [21,22]. Similarly, failure to differentiate between cooling methodologies may cloud our understanding of the mechanisms driving performance enhancement. Such differentiation [26] may be of use in future studies that plan to tease out the differences between combined methodologies, and for practitioners who require variety in cooling strategies dependent upon athletes’ competitive environments and regulations.

Of the chosen methods, combining cooling timings demonstrated the greatest effect on rectal temperature (Small: −0.59; −0.90 to 0.28); however, the breadth of confidence interval suggests variability in the rate at which lowering of rectal temperature [30] takes place, and subsequent accumulation of heat [30,31,32] across the exercise duration. This variability likely occurs at an individual level, as all included trials were carried out in conditions exceeding the temperature at which metabolic heat production outweighs thermal transfer [20]. The intensity of precooling [31] and the subsequent rate of increase in rectal temperature following precooling [30,31] may contribute to the efficacy of precooling strategies.

It is important to note that the ice slurries used in the majority of included precooling studies contained carbohydrate [11,33,34,35,36], which may have conveyed a physiological advantage beyond precooling alone, although it is acknowledged that the main purpose of carbohydrate in these beverages was to act as an antifreeze [37,38]. The amounts of carbohydrate ingested in each study are in congruence with current recommendations for exercise lasting up to 2 h (≤120 g) [39] and so may have elicited ergogenic effects in these investigations.

Percooling provided a small beneficial improvement in time trial performance (−0.21; −0.48 to 0.05), with all studies reporting a mean reduction in time trial performance, despite the use of ingested and topical methods [5,11,35,36,40,41]. Differences in cooling methodology (ingested; topical) may evoke distinct responses, attributable to differing underpinning mechanisms, despite achieving a uniform effect upon time trial performance when applied throughout the exercise bout. Ingested percooling methods may initially impart perceptual feelings of freshness through stimulation of the cold and menthol sensitive TRPM8 receptors [42,43]. Strategies containing menthol improved time trial performance to a greater extent than non-menthol containing counterparts in a temperature dependent manner [12,13]. Menthol has also demonstrated improvements in time trial performance and time to exhaustion when used as a mouth rinse [4,11], suggesting that the refreshing sensation or perceptual cooling experienced by an athlete may further enhance the wider physiological effects observed in other percooling studies, especially when isolated to the oral cavity [32].

Ingested cooling strategies may also act as a thermal buffer, attenuating a rise in rectal temperature, whereby gastrointestinal temperature is reduced prior to exercise commencing (heat sink) [12,14]. Furthermore, it is hypothesised that ice ingestion may cause a mismatch between core and brain temperatures, where the brain perceives the lower local temperature as a greater heat sink than that which has been induced at the core [14,38]. Greater metabolic heat production is then permitted due to this perceived difference, evidenced by the ‘overshoot’ in rectal temperature seen at exercise termination in some ice ingestion studies [30,37]. Percooling, on the other hand, may permit an initial beneficial rise in core temperature and resultant physiological responses prior to a subsequent dampening of any potentially limiting effects. Percooling may also alleviate subjective thermal measures over a more prolonged duration compared to precooling because of repeated exposures to cold stimuli. This cannot be confirmed by the included studies due to the difference in time trial durations between precooling and percooling conditions. Ingested cooling strategies consumed across the entire exercise window therefore strike a balance between attenuating physiological symptoms and perceptual sensations, especially when combined with menthol at lower temperatures [12,13].

If athletes cool the oral cavity during exercise, using cool liquid, ice, or even menthol, as in the works of Riera [12] and Tran Trong [13], cold receptors are stimulated in the oral cavity, conferring a hedonic effect and possibly satiating thirst [44,45]. Satiating thirst may also reduce the likelihood of gastrointestinal distress associated with ingesting large volumes of liquid, particularly when running [46]. The role of menthol in facilitating ingested cooling methodologies also warrants further investigation [32].

Topical percooling lowers skin temperature, inducing cutaneous vasoconstriction and increasing the temperature gradient between the skin and the external environment in hot conditions [47]. This mechanism permits convective and radiative heat exchange up to temperatures of 36 °C [47], beyond which evaporative cooling becomes the main method of body temperature regulation. Dry, windy conditions that promote convection and evaporation [48] are required for topical cooling to be most effective. The included topical percooling studies [5,49] present practical ways of cooling athletes that are less cumbersome than typical precooling strategies, namely pouring cold water over the body and the application of cold towels. Both methods could be easily transported in a cool box and kept roadside or trackside. The pouring of cold water is especially valid and practical, with many athletes already doing so in competition [40,48].

Skin wetness may also be important in cooling and is influenced by factors pertinent to athlete comfort during endurance exercise in the heat, such as humidity and airflow over the skin [49]. In the absence of airflow over the skin, topical and combined methodologies applied within lab conditions improve thermal comfort and sensation by providing targeted stimuli that aggressively reduce local skin temperatures [1]. Some topical methods may promote skin wetness (cold water and/or towels) and therefore facilitate evaporative cooling, whereas others (ice vests) stimulate large, cold-sensitive areas such as the chest and back [31,49,50,51], and reduce skin temperature very quickly, all important factors in improving thermal perceptions [51].

Although no positive effects on time trial performance or power output were noted following topical or combined strategies, performance did not worsen either ([5,34,35]; Table 2). There may be occasions where an attenuation of an athlete’s perception of thermal state is beneficial, provided performance does not deteriorate (e.g., a domestique in the Tour de France, who must maintain a consistent level of performance over 21 days of riding, in rapidly changing thermal circumstances, with his performance tasks altering depending on the needs and strategies of his team day by day).

We found combining topical and ingested cooling methods to only have a trivial effect upon time trial performance with a broad confidence interval (−0.07; −0.44 to 0.29), supported by an expectedly trivial change in power output (0.02; −0.39 to 0.44). Combined cooling methods do, however, markedly lower rectal temperature whilst also improving thermal comfort and sensation, although they may inhibit physiological processes facilitative to endurance performance in the heat, such as increased vasodilation, muscular or skin blood flow, and sweating [52]. The moderate reduction in rectal temperature seen when combining methodologies likely results in an insufficient temperature gradient between the core and skin, dampening the performance enhancing effects either treatment would promote in isolation. The breadth of the confidence intervals around the trivial performance effects of combined cooling methodologies may be explained in part by the individual, and regional variation in these physiological responses, as well as the heterogeneity of study designs [32,53,54].

The range of observed responses, as evidenced through broad confidence intervals, suggest that the timing and methodology employed can affect athletes’ performances differently, and, more importantly, that differing strategies may target different mechanisms (i.e., a reduction in either perceived [4,11,32,41] or physiological thermal load [11,34,35,36,41,55], or both [12,13]). Each targeted mechanism(s) likely possesses differing levels of intra- and inter-individual variability, and this may further vary between investigations, as per Figure 2 and Table 2. Quantifying the coefficient of variation in athletes’ performances and associated measures (e.g., thermal comfort or sensation) is an important step in assessing the efficacy of an intervention. If an intervention produces a change that is greater than the coefficient of variation observed in an individual or group, it can be deemed to have had an effect. Several papers provide a good starting point for this analysis in cycling [23], running [53,54,55,56], and triathlon [57], and Atkinson and Nevill [58] provide a working example for the practitioner.

Finally, although beneficial in acute settings, little is known about the long-term application of cooling interventions in the absence of heat acclimation. Repeated exposure to extremes of temperature may be detrimental to long-term health [27], and if cooling strategies are employed to repeatedly facilitate such exposure over the course of a season or training cycle then athlete health should be monitored accordingly.

## 5. Conclusions

We found that ingested cooling methodologies show ecologically important small improvements in time trial performance when applied before and during endurance exercise bouts (Figure 2; Table 2). Improvements in time trial performance and power output may be attributable to differing mechanisms (perceptual or physiological cooling) depending upon the cooling strategy being administered [16]; further elucidation of these mechanisms and their effects upon performance and long-term health is still required [59,60]. Carbohydrate provision may be a confounding but contributory factor with respect to the investigation of cooling strategies as a means of performance enhancement.

When choosing a cooling strategy, we urge practitioners to consider the strategy’s effects holistically, assessing athletes’ perceptual and physiological responses to cooling in training prior to competition. Optimal frequency and timing of cooling strategies is likely a convergence of athletes’ responses to cooling interventions and sport-specific statutory limitations (e.g., number of feed stations). Simply providing athletes with cool or cold water before and during events allows for athletes to ingest, swill, or pour the liquid over themselves, and therefore is a useful first step for providing cooling interventions. If practitioners can provide athletes with ice slurries for ingestion, this would likely further improve performance by ameliorating thermal comfort and sensation, and an attenuation of core temperature—the addition of menthol may support these effects.

## Figures and Tables

**Table 1:** Details of studies included for meta-analysis including participant number, timings, methods of cooling, exercise modality and study outcomes.

Author	Participants	Timing	Intervention	Modality	Outcomes
Ross et al., 2011	11	Precooling	Ice	Cycling	TT, PO, T_rec_, TC
Ross et al., 2012	12	Precooling	Ice + T, Ice + G + T	Cycling	TT, PO, RPE, TC
Muñoz et al., 2012	10	Percooling	OR, EXC, EXC + OR	Running	TT, T_rec_, TC, RPE
Stanley et al., 2010	10	Percooling	Ice, COOL	Cycling	TT, PO, T_rec_
Stevens et al., 2013	9	Percooling	Ice	Triathlon/Running	TT, T_rec_, RPE, TC
Stevens et al., 2015	11	Precooling/Percooling	Ice, M	Running	TT, T_rec_, RPE, TS
Stevens et al., 2017	9	Percooling	M	Running	TT, T_rec_, RPE, TS
Riera et al., 2014	12	Combined	N, N + M, COOL, COOL + M, Ice, Ice + M	Cycling	TT, TC, TS, RPE
Tran Trong et al., 2015	10	Combined	N + M, COOL + M, Ice + M	Cycling/Running	TT, TC, TS, RPE
Schulze et al., 2015	7	Combined	Ice, PC + Ice	Cycling	TT, PO, T_rec_, TC, TS

Intervention Methodologies: COOL: cool liquid ingestion; EXC: external cooling via pouring cold water; G: glycerine; Ice: ice slurry ingestion; N: ambient temperature water; M: menthol; OR: oral rehydration; T: iced towels applied to participants. Outcome Variables: TT: time trial performance; PO: power output; RPE: rating of perceived exertion; T_rec_: rectal temperature; TC: thermal comfort; TS: thermal sensation.

**Table 2:** Change in time trial performance of studies included for meta-analysis including timings and methods of cooling.

Author	Timing	Intervention	∆ Performance (s)
Ross et al., 2011	Precooling	Ice	−66.0 ± 29.4
Ross et al., 2012	Precooling	Ice + T	−18.6 ± 28.8
-	-	Ice + G + T	0.0 ± 1.2
Muñoz et al., 2012	Percooling	OR	−60.0 ± 81.0
-	-	EXC	−48.0 ± 85.2
-	-	EXC + OR	−63.0 ± 52.2
Stanley et al., 2010	Percooling	Ice	−33.6 ± 60
Stevens et al., 2013	Percooling	Ice	−72.0 ± 18.0
Stevens et al., 2015	Precooling	Ice	18.0 ± 12.0
-	Percooling	M	−42.0 ± 6.0
Stevens et al., 2017	Percooling	M	−36.0 ± 6.0
Riera et al., 2014	Combined	N + M	−49.8 ± 33.6
-	-	COOL	36 ± 139.8
-	-	COOL + M	−162.6 ± 39.0
-	-	Ice	−121.2 ± 12.6
-	-	Ice + M	−232.8 ± 51.0
Tran Trong et al., 2015	Combined	COOL + M	−136.2 ± 252.0
-	-	Ice + M	−283.2 ± 232.8
Schulze et al., 2015	Combined	Ice	−23.4 ± 0.0
-	-	Ice + T	4.8 ± 6.0

Intervention Methodologies: COOL: cool liquid ingestion; EXC: external cooling via pouring cold water; G: glycerine; Ice: ice slurry ingestion; N: ambient temperature water; M: menthol; OR: oral rehydration; SC: scalp cooling; T: iced towels applied to participants.
